# Flash’O real-world evidence programme – Attitude and practices toward the use of omega-3 FA by physicians from Middle East Countries

**DOI:** 10.1097/MD.0000000000035416

**Published:** 2023-10-06

**Authors:** Abdulhalim Jamal Kinsara, Hani Sabbour

**Affiliations:** a Ministry of National Guard health affairs, King Saud bin Abdulaziz University for Health Sciences, COM-WR, King Abdullah International Research center, Riyadh, Saudi Arabia; b Heart and Vascular Institute, AL Maryah Island, Abu Dhabi Global Market Square, Abu Dhabi, United Arab Emirates.

**Keywords:** DHA, EPA, omega-3 fatty acids, physicians, real-world clinical practice, survey

## Abstract

The Flash’O project was designed to provide insights into the current use of prescription omega-3 and their perceived benefits by physicians in real-world clinical practice, in Russia, Saudi Arabia, Thailand, and Gulf countries, and to determine the adherence of physicians to dyslipidemia management guidelines. The present study focuses on Flash’O’s process and results in Middle East countries.

A total of 338 physicians and specialists completed the online questionnaire. Most responding physicians were male (91.7%), general practitioners (42.6%) with more than 5 years of seniority (80.4%) and saw more than 50 patients a week (71.5%). Most surveyed physicians (64.2%) reported using guidelines in their daily practice for the management of their patients with dyslipidemia. They mostly followed national guidelines (68.6%). American or European ones were less commonly used.

Responding physicians thought that omega-3 supplementation could be more beneficial in all types of dyslipidemia, except high non- hight density lipoproteins, and for patients suffering from obesity, type 2 diabetes mellitus, acute coronary syndrome with ST-segment elevation myocardial infarction and high cardiovascular diseases risk (score ≥ 5% and < 10%), but less beneficial in chronic kidney disease. Respondents recommended omega-3 to their patients mainly after statin treatment in patients with dyslipidemia and for the treatment of dyslipidemia.

This survey confirmed that omega-3 fatty acids are at the heart of the cardiovascular medical strategy.

## 1. Introduction

Despite significant advances in their prevention and treatment, cardiovascular diseases remain the leading cause of mortality worldwide. Ischemic heart disease and stroke were the first and second leading global causes of death in 2019,^[1]^ accounting respectively for 16% and 11% of 55.4 million deaths worldwide. The largest increase in deaths since 2000 is reported for ischemic heart disease, rising by more than 2 million to 8.9 million deaths in 2019.^[1]^ In Kuwait, for instance, the estimated death rate per 100,000 population in 2019 was 55.3 for ischemic heart disease and 14.2 for stroke.^[2]^ It has increased by 24.4% and 10.6% respectively since 2005.^[2]^

The omega-3 long-chain polyunsaturated fatty acids (PUFA) family has been the subject of intense investigation since their abundance in the diet of Greenland Inuit people, which contains large amounts of fish and other marine animals, was associated with a low incidence of ischemic heart disease in the 1970s.^[3,4]^ Omega-3 fatty acids (FA) are incorporated into membranes of all tissues, in particular, in the brain, retina, and myocardium.^[5]^ Omega-3 FA have multiple biological effects and play a significant role in the process of blood coagulation, in inflammation, regulation of blood vessel contractility and proper brain and eye retina functioning.^[6,7]^ They play key roles in growth and development, and also the maintenance of optimal health across all age groups.^[8,9]^ Eicosapentaenoic acid (EPA) and docosahexaenoic acid (DHA), major representatives of this lipid class, are concentrated in oils from marine fish, krill, and microalgae. In the Global Burden of Disease Study 2017, evaluating the consumption of major foods and nutrients across 195 countries between 1990 and 2017 and the impact of their suboptimal intake, diets low in seafood omega-3 FA have been ranked as the 6th most important dietary risk factor.^[10]^ A total of 1.5 million deaths and 33 million disability-adjusted life-years worldwide were attributable to this deficiency. The mortality risk decreases by 10% to 30% with each 100 mg increment in dietary EPA and DHA intake.^[10]^ It has been estimated that <20% of the world’s population consumes the recommended minimum of 250 mg/day of omega-3 FA (EPA and DHA).^[11]^

Cardiovascular effects of omega-3 FA have been studied extensively in vitro and in vivo, in animal experiments, controlled feeding studies, epidemiological studies, and randomized controlled trials (RCTs) in humans. Many clinical studies, examining different patient populations and using different forms and dosages, demonstrated the beneficial health effects of omega-3 FA on cardiovascular outcomes.^[12–17]^ Several guidelines have been issued which recommend omega-3 FA supplementation. These include, for example, Food and Agriculture Organization’s guidelines on an international level^[18]^ and European Food Safety Authority’s guidelines for Europe.^[19]^ Highly purified long-chain omega-3 FA, available as prescription-only medications, are approved for the treatment of severe hypertriglyceridemia and for the secondary prevention of major cardiovascular events after myocardial infarction by different health agencies.^[20]^ Prescription-grade omega-3 FA supplements are different from omega-3 PUFA dietary supplements.^[21]^ Prescription omega-3 FA products have greater EPA/DHA concentrations, are highly purified, have clinical evidence and approved clinical indications, and are subject to the rigorous regulatory standards required for medications. In contrast, safety issues including variable content of EPA and/or DHA, inconsistencies with labeled quantities, poor product quality, and impurities such as oxidized fatty acids, toxins (e.g., mercury), and unwanted ingredients such as cholesterol and saturated fat have been documented for omega-3 dietary supplements.^[5,22]^

While multiple RCTs have been performed to evaluate the effects of omega-3 FA, little information is available about physicians’ attitude and practices toward the use of omega-3 FA. Two previous studies conducted among primary care physicians in the United States^[23]^ and cardiologists in Karachi, Pakistan^[24]^ showed that despite favorable attitudes toward diet and adequate knowledge of the benefits of use of omega-3 FA, they infrequently prescribed them as supplement or in diet to CVD patients.

The Flash’O project was designed to provide insights into the current use of prescription omega-3 and their perceived benefits by physicians in real-world clinical practice, especially in Russia, Saudi Arabia, Thailand, and Gulf countries, and to determine the adherence of physicians to dyslipidemia management guidelines. The present study focuses on Flash’O’s process and results in Middle East countries. The main goals were to obtain Middle East’s physicians opinions regarding the areas where patients benefit most from using omega-3 and to point out benefits of omega-3 in the management of patients with dyslipidemia.

## 2. Methods

### 2.1. Study objectives, study design and participants

#### 2.1.1. Study design.

The medical research was an online survey consisting of a self-administered questionnaire. The survey was conducted in Saudi Arabia and Gulf countries (Kuwait, United Arab Emirates, Qatar, and Bahrain). For Saudi Arabia and UAE, due to the COVID-19 pandemic the project was kick-of completely remotely through a dedicated online Webinar Session in October 2020. Around 500 participants were connected and reply in live to the online survey. Therefore, responses were collected during this moment, and the questionnaire didn’t need to be online for weeks.

Since the survey was not a clinical study, there were no formal study hypotheses and no formal calculation of sample size. It was planned to contact at least 1000 physicians to get around 300 respondents. The research aimed to collect data by using online questionnaire to approach physicians. Participating physicians had to read the information sheet which was sent in a separate file. The online questionnaire was sent through a link, send by the ambassadors. The link refers to an online platform where respondents had to subscribe, and then the first page was a privacy policy disclaimer.

Participating physicians had to confirm that they understood the purpose of this study and the process relevant to use of their personal information (or data) before completing the questionnaire.

The answers from all participating physicians were collected and analyzed in aggregate only. All the results were anonymous.

#### 2.1.2. Participants.

Physicians prescribing omega-3 in Saudi Arabia and Gulf countries (Kuwait, United Arab Emirates, Qatar, and Bahrain) were invited to take part in the survey. They were cardiologists, endocrinologists/diabetologists, nutritionists, nephrologists, general practitioners, or internal medicine specialists.

### 2.2. Questionnaire

A steering committee was constituted to lead the project, with 1 representative from each of the countries. Based on relevant literature reviews and clinical expertise of the steering committee members, a questionnaire was developed to allow assessment of omega-3 usage benefits. The questionnaire was in English to ensure appropriate understanding of the questions raised.

The questionnaire consisted of 4 main sections. The first section included questions about physician’s profile (country of practice, gender, medical specialty, number of years in practice, average number of patients seen per week). In the second part, physicians were asked whether they use guidelines for the management of patients with dyslipidemia and which ones are followed (national, American [American Heart Association/American College of Cardiology/National Lipid Association 2018; Department of Veterans Affairs 2014], American Diabetes Association “Standards of Medical Care in diabetes 2020,” and European [European Society of Cardiology/European Atherosclerosis Society 2019]). The third section consisted of 17 items related to the benefits of the use - evaluated using a scale ranging from 0 (limited benefit) to 10 (major benefit) - of omega-3 in several cardiovascular, kidney diseases, or metabolic disorders. The 17 items were: Acute coronary syndrome: ST-segment elevation myocardial infarction; Acute coronary syndrome: non-ST-segment elevation myocardial infarction; Acute coronary syndrome: unstable angina; Elevated CVD risk: High risk: Calculated SCORE ≥ 5% and < 10%; Elevated CVD risk: Very High risk: Calculated SCORE ≥ 10%; Chronic kidney disease, with mild to moderate renal impairment; Chronic kidney disease with severe renal impairment; Type 2 Diabetes Mellitus; Mixed Dyslipidemia: High triglycerides (TGs) – low hight density lipoproteins (HDL); Dyslipidemia: TGs: 200 to 500 mg/dL (2.3 to 5.7 mmol/L); Dyslipidemia: TGs > 500 mg/dL (>5.7 mmol/L); Dyslipidemia: High Non-HDL; Heart Failure; Obesity; Post myocardial infarction (<1 month); Post MI with Left Ventricular Ejection Fraction < 40%; and Stroke. The last section dealt with the frequency of use - scored using a scale from 0 (never)] to 6 (always) - in 3 situations: for secondary prevention of myocardial infarction, in patients with dyslipidemia, and after statin treatment in patients with dyslipidemia.

### 2.3. Statistical analysis

Results were expressed using standard descriptive statistics. Categorical variables were summarized using percentages (sections 1 and 2). Continuous variables (sections 3 and 4) were summarized using means and standard deviations. Benefits of the use of omega-3 were presented after exclusion of responses “do not know.” For the frequency of use of the omega-3, the answer “do not know” received a score of 0.

#### 2.3.1. Clustering opinions.

The first step aimed at reducing the information from the 17 questions about opinions into a reduced number of variables prior to run a cluster analysis based on the physician’s opinions regarding omega-3 in several cardiovascular, kidney diseases, or metabolic disorders. All data were taken into account, and a score of 0 was attributed when the answer was “do not know.” These 17 questions were first pooled into 1 cluster, and then clustered using the SAS procedure *proc varclus* involving an iterative clustering process as follows: if the variation explained by this cluster was <75%, the cluster was divided into 2 clusters. Then, for each cluster, if the variation explained by each cluster was inferior to 75%, each cluster was divided into 2 clusters. This process was reiterated until the variation explained by each cluster was higher than 75%. For each cluster of questions that was retained, a new score was derived as the mean of the score of the questions that composed the cluster.

#### 2.3.2. Clustering physicians based on their opinion.

A principal component analysis was performed on the clustered opinions previously obtained. Resulting principal components with an eigenvalue superior to 1 (or proportion superior to 10%) were retained and their values for each physician were recorded. Finally, these principal components obtained from the principal component analysis were used in the Ward’s hierarchical cluster analysis. The quality of the clustering (R-square) in function of each step of the clustering process was graphically assessed. The last maximal gap between 2 steps was used to determine the number of clusters. Once the number of clusters was determined, the position of the physicians was mapped on the 2 first principal components graph to visualize the physician clusters.

All data were analyzed using SAS software (Soladis Group, 160 bis Rue du Temple, 75003 Paris, France).

## 3. Results

Among the 402 physicians participating in the survey, 338 were included in the analysis. Among the physicians who participated to the survey, 308 were from Saudi Arabia. A total of 64 physicians were excluded from the analysis because their answer was “Do not know” to the 17 questions regarding the benefits of omega-3 in specific medical conditions (N = 53, 82.8%) or because no medical specialty was documented (N = 11, 17.2%).

### 3.1. Physician characteristics

Of the included physicians, 91.7% were male. Of the included physicians, most respondents were general practitioners (42.6%) followed by nutritionists (34.9%) and internal medicine specialists (17.5%). Thirty-eight-point four percentage of respondents had been in practice for more than 10 years, and 80.4% of the respondents had more than 5 years seniority. Most physicians (71.5%) reported seeing more than 50 patients per week, and 48.2% of physicians saw more than 100 patients per week. Descriptive statistics of physician characteristics are presented in Table 1.

**Table 1:** Physician characteristics.

		Overall N = 338
Female	n/N (%)	28/338 (8.3%)
Male	n/N (%)	310/338 (91.7%)
Medical specialty		
Cardiologist	n/N (%)	12/338 (3.6%)
Endocrinologist/diabetologist	n/N (%)	4/338 (1.2%)
General practitioner	n/N (%)	144/338 (42.6%)
Internal medicine	n/N (%)	59/338 (17.5%)
Nephrologist	n/N (%)	1/338 (0.3%)
Nutritionist	n/N (%)	118/338 (34.9%)
Seniority (yr)		
< 2 yr	n/N (%)	23/338 (6.8%)
2–4 yr	n/N (%)	43/338 (12.7%)
5–9 yr	n/N (%)	142/338 (42.0%)
10–14 yr	n/N (%)	87/338 (25.7%)
≥ 15 yr	n/N (%)	43/338 (12.7%)
Weekly activity (number of patients)		
≤ 10	n/N (%)	24/338 (7.1%)
11–30	n/N (%)	38/338 (11.2%)
31–50	n/N (%)	34/338 (10.1%)
51–80	n/N (%)	41/338 (12.1%)
81–100	n/N (%)	38/338 (11.2%)
>100	n/N (%)	163/338 (48.2%)

### 3.2. Use of guidelines

Most surveyed physicians (64.2%) reported the use of guidelines in their daily practice for the management of patients with dyslipidemia (Table 2).

**Table 2:** Guidelines use per specialty.

		Cardiologist	Endocrinologist Diabetologist	General Practitioner	Internal Medicine	Nephrologist	Nutritionist	Overall
		N = 12	N = 4	N = 144	N = 59	N = 1	N = 118	N = 338
Use of guidelines								
No	n/N (%)	0/12 (0.0%)	0/4 (0.0%)	47/140 (33.6%)	18/56 (32.1%)	0/1 (0.0%)	52/114 (45.6%)	117/327 (35.8%)
Yes	n/N (%)	12/12 (100.0%)	4/4 (100.0%)	93/140 (66.4%)	38/56 (67.9%)	1/1 (100.0%)	62/114 (54.4%)	210/327 (64.2%)
National†								
No	n/N (%)	8/12 (66.7%)	2/4 (50.0%)	24/93 (25.8%)	15/38 (39.5%)	0/1 (0.0%)	17/62 (27.4%)	66/210 (31.4%)
Yes	n/N (%)	4/12 (33.3%)	2/4 (50.0%)	69/93 (74.2%)	23/38 (60.5%)	1/1 (100.0%)	45/62 (72.6%)	144/210 (68.6%)
American (AHA/ACC 2013; Department of Veterans Affairs 2014)†								
No	n/N (%)	4/12 (33.3%)	3/4 (75.0%)	71/93 (76.3%)	26/38 (68.4%)	1/1 (100.0%)	49/62 (79.0%)	154/210 (73.3%)
Yes	n/N (%)	8/12 (66.7%)	1/4 (25.0%)	22/93 (23.7%)	12/38 (31.6%)	0/1 (0.0%)	13/62 (21.0%)	56/210 (26.7%)
American Diabetes Association ‘Standards of Medical Care in diabetes 2020 ‘†								
No	n/N (%)	10/12 (83.3%)	2/4 (50.0%)	67/93 (72.0%)	25/38 (65.8%)	1/1 (100.0%)	48/62 (77.4%)	153/210 (72.9%)
Yes	n/N (%)	2/12 (16.7%)	2/4 (50.0%)	26/93 (28.0%)	13/38 (34.2%)	0/1 (0.0%)	14/62 (22.6%)	57/210 (27.1%)
European (ESC/EAS 2019)†								
No	n/N (%)	5/12 (41.7%)	4/4 (100.0%)	80/93 (86.0%)	29/38 (76.3%)	1/1 (100.0%)	57/62 (91.9%)	176/210 (83.8%)
Yes	n/N (%)	7/12 (58.3%)	0/4 (0.0%)	13/93 (14.0%)	9/38 (23.7%)	0/1 (0.0%)	5/62 (8.1%)	34/210 (16.2%)

† in physicians using guidelines.

Among physicians using guidelines, most of them (68.6%) stated that they use national ones, but not the American or European guidelines (Table 2).

Analysis per specialty and country showed that European and American guidelines were followed by most cardiologists (58.3% and 66.7% respectively), while national ones were used by general practitioners (74.2%), internal medicine specialists (60.5%), nephrologists (100%) and nutritionists (72.6%).

### 3.3. Perceived benefits of omega-3 in specific medical conditions

Overall, most of the respondents (76.4%) think that omega-3 are more efficient in patients older than 70 years old, with the highest percentage regardless the specialty, except the nephrologist who does not know if the use of Omega-3 is more beneficial in patients older than 70 years old (Table 3).

**Table 3:** Opinions regarding the benefit of Omega-3 in patients older than 70 years, overall and per medical specialty.

		Cardiologist	Endocrinologist Diabetologist	General Practitioner	Internal Medicine	Nephrologist	Nutritionist	Overall
		N = 12	N = 4	N = 144	N = 59	N = 1	N = 118	N = 338
More efficient								
Yes	n/N (%)	6/11 (54.5%)	3/4 (75.0%)	112/143 (78.3%)	45/56 (80.4%)	0/1 (0.0%)	87/116 (75.0%)	253/331 (76.4%)
No	n/N (%)	1/11 (9.1%)	1/4 (25.0%)	8/143 (5.6%)	6/56 (10.7%)	0/1 (0.0%)	15/116 (12.9%)	31/331 (9.4%)
Don’t know	n/N (%)	4/11 (36.4%)	0/4 (0.0%)	23/143 (16.1%)	5/56 (8.9%)	1/1 (100.0%)	14/116 (12.1%)	47/331 (14.2%)
ND	n	1	0	1	3	0	2	7

**Figure 1.**
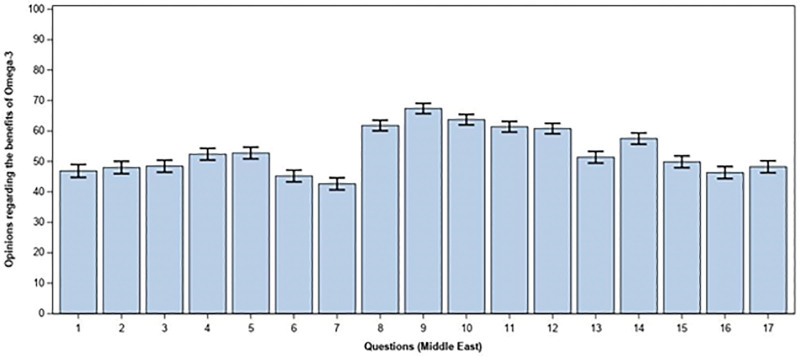
Opinions regarding the benefits of Omega-3 in specific medical conditions. (A) Acute coronary syndrome: ST-segment elevation myocardial infarction. (B) Acute coronary syndrome: non-ST-segment elevation myocardial infarction. (C) Acute coronary syndrome: unstable angina. (D) Elevated CVD risk: High risk: Calculated SCORE ≥ 5% and < 10%. (E) Elevated CVD risk: Very High risk: Calculated SCORE ≥ 10%. (F) Chronic kidney disease, with mild to moderate renal impairment. (G) Chronic kidney disease with severe renal impairment. (H) Type 2 Diabetes Mellitus. (I) Mixed Dyslipidemia: High TGs – low HDL. (J) Dyslipidemia: TGs: 200–500 mg/dL (2.3 to 5.7 mmol/L). (K) Dyslipidemia: TGs > 500 mg/dL (>5.7 mmol/L). (L) Dyslipidemia: High Non-HDL. (L) Heart Failure. (M) Obesity. (N) Post myocardial infarction (<1 month). (O) Post MI with Left Ventricular Ejection Fraction < 40%. (P) Stroke. HDL = hight density lipoproteins, TGs = triglycerides.

Physicians from Middle East countries estimated that omega-3 usage could have beneficial effects (means > 70) in all types of dyslipidemia, except high non-HDL, and for patients suffering from obesity, type 2 diabetes mellitus, acute coronary syndrome with ST-segment elevation myocardial infarction and high CVD risk (score ≥ 5% and < 10%) (Fig. 1).

While looking at specialties, omega-3 is perceived as beneficial for all types of acute coronary syndrome by cardiologists, internal medicine specialists and the nephrologist, for ST-segment elevation myocardial infarction by general practitioners and nutritionists, and for unstable angina by nutritionists. It is not perceived as beneficial (means ≤ 64) by endocrinologists/diabetologists. Likewise, endocrinologist/diabetologists don’t think that omega-3 is beneficial for elevated CVD risk, type 2 diabetes mellitus and all types of dyslipidemia, while cardiologists, internal medicine specialists, the nephrologist (except for high CVD risk) and nutritionists think that it is. However, general practitioners believe that omega-3 FA could only have beneficial effects for high TG – low HDL and for TG values between 200 and 500 mg/dL. Concerning chronic kidney disease (CKD), endocrinologists/diabetologists, general practitioners, internal medicine specialists and nutritionists do not think that w-3 PUFA can have beneficial effects, but cardiologists think that it can. Moreover, these FA are perceived as effective by internal medicine specialists for heart failure and as ineffective by cardiologists, endocrinologist/diabetologist and the nephrologist. For obesity, cardiologists, internal medicine specialists and nutritionists think that omga-3 can have beneficial effects but endocrinologists/diabetologists don’t, and for post myocardial infarction, cardiologists and endocrinologists/diabetologists don’t think that it can be effective while nutritionists find it beneficial for post myocardial infarction (<1 month). Finally, these FA are perceived as effective by cardiologists, endocrinologists/diabetologists and internal medicine specialists for stroke (Table 4).

**Table 4:** Opinions regarding the benefits of omega-3 in specific medical conditions, overall and per medical specialty (scale: 0-100).

	Cardiologist	Endocrinologist Diabetologist	General Practitioner	Internal Medicine	Nephrologist	Nutritionist	Overall
	N = 12	N = 4	N = 144	N = 59	N = 1	N = 118	N = 338
Acute coronary syndrome: ST-segment elevation myocardial infarction							
n (nmiss)	10 (2)	2 (2)	94 (50)	37 (22)	1 (0)	74 (44)	218 (120)
m ± SD	75.5 ± 25.8	57.0 ± 22.6	71.4 ± 22.9	71.7 ± 22.8	92.0±.	74.5 ± 21.5	72.7 ± 22.4
Acute coronary syndrome: non-ST-segment elevation myocardial infarction							
n (nmiss)	10 (2)	2 (2)	99 (45)	38 (21)	1 (0)	82 (36)	232 (106)
m ± SD	77.7 ± 24.6	44.5 ± 4.9	69.3 ± 22.6	72.3 ± 21.7	76.0±.	69.2 ± 21.9	69.9 ± 22.2
Acute coronary syndrome: unstable angina							
n (nmiss)	10 (2)	3 (1)	98 (46)	41 (18)	1 (0)	84 (34)	237 (101)
m ± SD	73.2 ± 25.7	41.0 ± 18.0	66.1 ± 23.5	72.4 ± 21.2	77.0±.	71.3 ± 20.4	69.1 ± 22.3
Elevated CVD risk: High risk: Calculated SCORE ≥ 5% and < 10%							
n (nmiss)	10 (2)	4 (0)	107 (37)	44 (15)	1 (0)	86 (32)	252 (86)
m ± SD	76.3 ± 26.1	40.8 ± 21.0	68.1 ± 20.6	71.3 ± 23.9	67.0±.	73.2 ± 18.2	70.3 ± 20.9
Elevated CVD risk: Very High risk: Calculated SCORE ≥ 10%							
n (nmiss)	11 (1)	4 (0)	111 (33)	39 (20)	1 (0)	92 (26)	258 (80)
m ± SD	79.4 ± 24.2	46.0 ± 26.0	65.6 ± 23.6	75.3 ± 20.5	71.0±.	70.6 ± 21.7	69.1 ± 22.9
Chronic kidney disease, with mild to moderate renal impairment							
n (nmiss)	10 (2)	2 (2)	102 (42)	40 (19)	1 (0)	82 (36)	237 (101)
m ± SD	73.8 ± 24.6	29.0 ± 8.5	62.8 ± 23.4	69.0 ± 23.0	68.0±.	64.0 ± 24.7	64.5 ± 23.9
Chronic kidney disease with severe renal impairment							
n (nmiss)	9 (3)	3 (1)	95 (49)	43 (16)	1 (0)	78 (40)	229 (109)
m ± SD	74.0 ± 25.6	23.0 ± 21.0	63.2 ± 24.8	60.9 ± 28.4	66.0±.	64.0 ± 26.5	62.9 ± 26.3
Type 2 diabetes mellitus							
n (nmiss)	11 (1)	3 (1)	126 (18)	48 (11)	1 (0)	104 (14)	293 (45)
m ± SD	86.2 ± 16.0	36.0 ± 5.6	69.1 ± 22.3	70.6 ± 26.3	75.0±.	73.7 ± 20.8	71.3 ± 22.6
Mixed dyslipidemia: High TG – low HDL							
n (nmiss)	11 (1)	4 (0)	126 (18)	49 (10)	1 (0)	109 (9)	300 (38)
m ± SD	90.1 ± 15.5	55.5 ± 26.1	73.5 ± 20.8	78.3 ± 22.2	89.0±.	76.9 ± 21.5	75.9 ± 21.4
Dyslipidemia: TG: 200-500 mg/dL (2.3 to 5.7 mmol/L)							
n (nmiss)	12 (0)	4 (0)	128 (16)	49 (10)	1 (0)	101 (17)	295 (43)
m ± SD	79.8 ± 28.6	53.3 ± 22.0	70.2 ± 21.7	74.5 ± 24.5	86.0±.	75.8 ± 19.5	73.0 ± 21.9
Dyslipidemia: TG > 500 mg/dL (>5.7 mmol/L)							
n (nmiss)	12 (0)	4 (0)	126 (18)	46 (13)	1 (0)	100 (18)	289 (49)
m ± SD	84.6 ± 21.8	58.0 ± 30.0	69.7 ± 21.9	73.2 ± 23.5	100.0±.	72.5 ± 19.6	71.8 ± 21.7
Dyslipidemia: High non-HDL							
n (nmiss)	12 (0)	4 (0)	125 (19)	50 (9)	1 (0)	103 (15)	295 (43)
m ± SD	81.3 ± 20.6	39.0 ± 28.1	65.8 ± 24.1	72.3 ± 20.1	87.0±.	72.7 ± 20.8	69.7 ± 22.7
Heart failure							
n (nmiss)	10 (2)	3 (1)	110 (34)	43 (16)	1 (0)	89 (29)	256 (82)
m ± SD	64.0 ± 25.8	30.0 ± 27.0	68.0 ± 22.5	70.0 ± 22.4	64.0±.	68.4 ± 24.0	67.9 ± 23.3
Obesity							
n (nmiss)	11 (1)	2 (2)	119 (25)	46 (13)	1 (0)	97 (21)	276 (62)
m ± SD	73.1 ± 18.3	48.5 ± 37.5	68.4 ± 22.6	72.6 ± 22.0	65.0±.	72.0 ± 23.2	70.4 ± 22.6
Post myocardial infarction (<1 mo)							
n (nmiss)	12 (0)	2 (2)	107 (37)	45 (14)	1 (0)	82 (36)	249 (89)
m ± SD	60.8 ± 30.7	49.5 ± 29.0	66.3 ± 21.9	68.6 ± 20.5	65.0±.	70.5 ± 23.7	67.7 ± 22.8
Post MI with left ventricular ejection fraction < 40%							
n (nmiss)	12 (0)	3 (1)	102 (42)	45 (14)	1 (0)	74 (44)	237 (101)
m ± SD	62.5 ± 32.4	32.7 ± 34.0	65.4 ± 23.7	65.3 ± 24.6	65.0±.	69.4 ± 22.3	66.1 ± 24.2
Stroke							
n (nmiss)	11 (1)	3 (1)	107 (37)	47 (12)	1 (0)	76 (42)	245 (93)
m ± SD	70.8 ± 25.5	70.0 ± 17.3	66.3 ± 23.0	70.1 ± 23.6	66.0±.	64.1 ± 25.4	66.6 ± 23.9

HDL = hight density lipoproteins.

### 3.4. Frequency of use of omega-3

On average, physicians reported that they often recommend the use of omega-3 FA to their patients for treatment of dyslipidemia and after statin treatment in patients with dyslipidemia (mean of 4.4 for both) (Table 5). The mean was lower for secondary prevention of myocardial infarction (3.9). For treatment of dyslipidemia, the highest frequency of use was reported by nephrologists (mean of 5), nutritionists and endocrinologists/diabetologists (4.5) and general practitioners (4.3). After statin treatment in patients with dyslipidemia, omega-3 were mostly prescribed by nutritionists and internal medicine specialists (4.5 each), followed by general practitioners (4.3) and cardiologists (4.1).

**Table 5:** Frequency of use of omega-3 overall and per specialty (scale: 0–6).

	Cardiologist	Endocrinologist Diabetologist	General Practitioner	Internal Medicine	Nephrologist	Nutritionist	Overall
	N = 12	N = 4	N = 144	N = 59	N = 1	N = 118	N = 338
For secondary prevention of myocardial infarction							
n (nmiss)	12 (0)	4 (0)	144 (0)	59 (0)	1 (0)	118 (0)	338 (0)
m ± SD	3.7 ± 2.0	2.5 ± 2.9	4.0 ± 1.4	4.1 ± 1.3	4.0±.	3.8 ± 1.6	3.9 ± 1.5
(Min;max)	(0.0;6.0)	(0.0;5.0)	(0.0;6.0)	(0.0;6.0)	(4.0;4.0)	(0.0;6.0)	(0.0;6.0)
Median	4.0	2.5	4.0	4.0	4.0	4.0	4.0
For treatment of dyslipidemia							
n (nmiss)	12 (0)	4 (0)	144 (0)	59 (0)	1 (0)	118 (0)	338 (0)
m ± SD	3.7 ± 2.0	2.5 ± 2.9	4.0 ± 1.4	4.1 ± 1.3	4.0±.	3.8 ± 1.6	3.9 ± 1.5
(Min;max)	(0.0;6.0)	(0.0;5.0)	(0.0;6.0)	(0.0;6.0)	(4.0;4.0)	(0.0;6.0)	(0.0;6.0)
Median	4.0	2.5	4.0	4.0	4.0	4.0	4.0
After statin treatment in patients with dyslipidemia							
n (nmiss)	12 (0)	4 (0)	144 (0)	59 (0)	1 (0)	118 (0)	338 (0)
m ± SD	3.7 ± 2.0	2.5 ± 2.9	4.0 ± 1.4	4.1 ± 1.3	4.0±.	3.8 ± 1.6	3.9 ± 1.5
(Min;max)	(0.0;6.0)	(0.0;5.0)	(0.0;6.0)	(0.0;6.0)	(4.0;4.0)	(0.0;6.0)	(0.0;6.0)
Median	4.0	2.5	4.0	4.0	4.0	4.0	4.0

## 4. Discussion

This survey was conducted to obtain physicians from Middle East countries’ opinions regarding the benefits of use of omega-3, especially in the management of patients with dyslipidemia.

A substantial number of physicians participated in this survey and 338 questionnaires could be included in the analysis. Respondents consisted of general practitioners and different specialists (cardiologists, endocrinologists/diabetologists, nutritionists, nephrologists, general practitioners, and internal medicine specialists) prescribing omega-3 supplements. These were experienced physicians (38.4% had been in practice for more than 10 years), and most respondents were general practitioners, followed by nutritionists and internal medicine specialists, which represent a mixture of nephrologists, cardiologists and endocrinologists.

Overall, there were more men than women (91.7% of respondents). This reflects the higher number of male health care professionals in the Middle East. Several societies, such as the American College of Cardiology/American Heart Association, National Lipid Association, Endocrine Society, the European Society of Cardiology and European Atherosclerosis Society have published guidelines for the classification and treatment of hypertriglyceridemia.^[25–29]^ The majority of surveyed physicians reported using guidelines in their daily practice for the management of dyslipidemia. National guidelines were the most commonly used.

This survey showed that omega-3 FA were seen as more beneficial in patients older than 70 years by 76.4% of physicians. These results are consistent with those of a review from 2012 which shows that beneficial effects of n-3 PUFA on cognitive function are mainly observed in the developing (potentially omega-3 deficient) and aged or cognitively impaired populations and that it is less effective in young individuals and adults with adequate omega-3 intake.^[30]^

Importantly, the survey also showed that omega-3 use is perceived as effective in all types of dyslipidemia, except high non-HDL, and for patients suffering from obesity, type 2 diabetes mellitus, acute coronary syndrome with ST-segment elevation myocardial infarction and high CVD risk (score ≥ 5% and < 10%) (Fig. 1).

Endocrinologist/diabetologists don’t think that omega-3 is beneficial for all types of dyslipidemia, while cardiologists, internal medicine specialists, the nephrologist and nutritionists think that it is. Moreover, general practitioners believe that omega-3 FA could only have beneficial effects for high TG – low HDL and for TG values between 200 and 500 mg/dL. In clinical practice, physicians reported that they often prescribed omega-3 FA for treatment of dyslipidemia and after statin treatment in patients with dyslipidemia. Dyslipidemia is a major risk factor for atherosclerotic CVD - which includes ischemic heart disease and cerebrovascular diseases such as stroke - and for type 2 diabetes mellitus, but this risk factor is modifiable by lifestyle changes and pharmacological interventions.^[31]^ Dyslipidemia is very frequent in the countries surveyed. In fact, in an observational study conducted in 2007 in 6 Middle Eastern countries, the prevalence of dyslipidemia in patients presenting with acute coronary syndrome was 36% in the United Arab Emirates, 45% in Bahrain, 37% in Kuwait and 29% in Qatar.^[32]^ A higher dyslipidemia rate of 72.5% was reported in a study conducted in 2012 to 2013 in adults living in the Northern Emirates.^[33]^ There is strong evidence that omega-3/DHA/EPA can reduce plasma TG levels. Several clinical studies (EVOLVE, MARINE, ANCHOR, EVOLVE II), reported a significant reduction in serum TG levels up to 45%.^[34–37]^ A 2020 Cochrane review including 86 RCTs published before August 2019 and 162,796 participants confirmed that increasing omega-3 FA intake decreases serum TG by about 15% in a dose-dependent manner.^[38]^ Their use at pharmacological doses is recommended in several guidelines to lower high TG levels, in particular in statin-treated patients who, despite a reduction in LDL cholesterol, remain at high residual cardiovascular risk with elevated TG levels.^[28,29]^ The lowering effect of omega-3 FA on TG is thought to involve decreased hepatic lipogenesis, increased b-oxidation of fatty acids, inhibition of key enzymes involved in hepatic TG synthesis, and increased expression of lipoprotein lipase.^[39]^

Although no more recommended by European guidelines for the secondary prevention after MI, our survey showed that some physicians and more particularly cardiologists, internal medicine specialists, the nephrologist and nutritionists think that omega-3 FA have a beneficial effect in several types of CVD. In addition, they expected a benefit in patients at high and very high risk of CVD, and/or in patients with heart failure, depending on the specialty. Ischemic heart disease and stroke are the first and second causes of death in Saudi Arabia and their prevention is crucial. The efficacy of the omega-3 FA in reducing CVD risk remains controversial as different RCTs, metanalyses and systematic reviews found inconsistent results. Several large RCTs supported the benefit of their use in diet and as supplements in secondary prevention of CVD, such as the GISSI Prevenzione in patients with recent MI,^[12]^ the GISSI-HF in patients with chronic heart failure,^[14]^ the JELIS study in hypercholesterolemic patients treated with statins,^[13]^ and the REDUCE-IT trial in statin-treated patients with hypertriglyceridemia and established CVD or diabetes.^[17]^ In contrast, other RCTs yielded neutral results, in particular the OMEMI study in elderly patients with recent MI^[40]^ and the STRENGTH study in statin-treated patients at high cardiovascular risk.^[41]^ In 2 other studies, VITAL in healthy men aged > 50 years and women aged > 55 years^[42]^ and ASCEND in patients with diabetes without evidence of atherosclerotic CVD,^[43]^ the primary composite endpoint was not significantly reduced, but some individual cardiovascular endpoints were reduced with EPA and DHA supplementation. Discrepancies between study results may be explained by differences in omega-3 baseline status (high fish intake), treatment compliance and baseline risk, the heterogeneity in study designs and the use of primary composite end points, the timing of the initiation of omega-3 FA supplements, a low omega-3 supplementation dose, insufficient length of follow-up to see benefits, inadequate controls, underpowered statistical analyses, potential interference in the omega-3 mechanisms of action by modern CVD prevention and treatment (especially use of statins and statin doses), use of EPA alone or both EPA and DHA, the chemical form of omega-3 FA and their interaction with food which might affect their degree of absorption.^[44–46]^ Data from meta-analyses have also yielded mixed results. The 2020 Cochrane review found that omega-3 FA had little or no effect on cardiovascular deaths and events, stroke or arrhythmias, but may reduce coronary heart disease mortality and events.^[38]^ In contrast, other metanalyses selecting studies with EPA + DHA supplementation only (and not dietary advice) showed their high effectiveness in the prevention of coronary heart disease events and MI, and a protective effect for CVD events and MI increasing with dosage.^[46,47]^ In another global pooled analysis, blood omega-3 FA levels were inversely associated with risk for death from CVD.^[48]^ Omega-3 may reduce the risk of CVD through different mechanisms, including TG-lowering, membrane stabilization, anti-arrhythmic, antithrombotic, anti-atherosclerotic, and anti-inflammatory effects, improving endothelial function and lowering blood pressure.^[7,49]^

In this survey, cardiologists, internal medicine specialists and nutritionists also expected a benefit of omega-3 FA in metabolic disorders such as diabetes mellitus type 2 and obesity, which are well-known risk factors for CVD. Worldwide obesity has nearly tripled since 1975. In 2016, more than 1.9 billion adults, 18 years and older, were overweight. Of these, over 650 million were obese.^[50]^ It is also the case in the Middle East where prevalence of obesity increased from 12% to 20% between 1980 and 2015.^[51]^ Diabetes mellitus has entered the top 10 causes of death, following a significant percentage increase of 70% since 2000.^[1]^ In the Middle East, 14.6% suffered from diabetes in 2019.^[52]^ Hypertriglyceridemia is a common lipid abnormality in persons with visceral obesity, and type 2 diabetes.^[53]^ Individuals with overweight or obesity have significantly lower omega-3 index (proportion of EPA and DHA in erythrocyte cell membrane lipids) or omega-3 PUFA status than their healthy-weight counterparts.^[54,55]^ Given their cardioprotective, anti-inflammatory and hypotriglyceridemic properties, omega-3 FA may be an important adjunct to obesity management, especially by improving individual components of the metabolic syndrome.^[56]^ Proposed mechanisms by which omega-3 FA could improve adiposity and metabolic disorders include modulating lipid metabolism; regulating adipokines, such as adiponectin and leptin; alleviating adipose tissue inflammation; promoting adipogenesis and altering epigenetic mechanisms.^[56]^

Low beneficial effects were expected in CKD by most physicians (endocrinologists/diabetologists, general practitioners, internal medicine specialists and nutritionists). Kidney disease was the 10th leading cause of death in 2019.^[1]^ According to the Global Burden of Disease study 2017, the global prevalence of CKD was 9.1% (697.5 million cases) in 2017 in the world, with an increase by 29.3% between 1990 and 2017, due to aging of the world’s population.^[57]^ In the Middle East, data are very limited, but prevalence is presumed to be great.^[58]^ Thus, kidney disease has a major effect on global health as a direct cause of global morbidity and mortality, and because it is associated with an increased risk for CVD mortality and is a risk multiplier in patients with hypertension and diabetes.^[57]^ Since CKD causes a profound dysregulation of lipoprotein metabolism with important alterations in the lipid profile of CKD patients, such as high TG and low HDL cholesterol, Harper and Jacobson recommend omega-3 FA for patients with mixed dyslipidemia (if not at non-HDL goal) or very high triglyceridemia and CKD from stage 3.^[59]^ Despite significantly lower blood levels of omega-3 FA in CKD patients than in the general population, omega-3 FA supplementation is not routinely used in CKD patients.^[60]^ A recent meta-analysis including 60 RCTs and 4129 participants suggested that omega-3 FA supplementation may reduce cardiovascular death for participants on hemodialysis and may prevent end-stage kidney disease in participants with CKD not receiving renal replacement therapy. Omega-3 FA may lower the risk of progression to end-stage kidney disease in patients with CKD not receiving yet dialysis by reducing blood pressure and inflammation, and improving endothelial function and erythrocytes membrane fluidity (Saglimbene et al^[61]^, 2020) and may reduce the inflammation in CKD patients on dialysis.^[60]^

The present paper describes a declarative study. It is based on voluntary answers from practitioners’ experience. In addition, data were analyzed based on a sample of physicians. Therefore, the results may not be representative of all specialties in Middle East countries, especially while most respondents were from Saudi Arabia. Moreover, there were only 4 endocrinologists/diabetologists and 1 nephrologist. Thus, their answers are not sufficient to make reliable conclusions regarding these specialties. However, the number of participants being high, overall results can be considered reliable.

## 5. Conclusion

In clinical practice, a beneficial effect of prescription-grade omega-3 FA is expected by physicians from the Middle East in various medical conditions, mainly related to atherogenic dyslipidemia, but also to metabolic disorders, acute coronary syndrome and high CVD risk. The TG-lowering effect of omega-3 FA has been documented in many RCTs, and responding physicians often prescribed them for secondary prevention of myocardial infarction and after statin treatment in patients with dyslipidemia. However, the survey shows that there are differences in the use of omega-3 depending on the indications. In fact, there are many different recommendations concerning Omega-3, and we can observe that physicians have created their own guidelines according to their practice and specialty. This survey confirmed that prescription-grade omega-3 FA are at the heart of the cardiovascular medical strategy.

## Acknowledgements

The authors would like to thank the ambassadors of Thailand, Korrakot Pornchaichanakit, Nipawan Waisayanand, Panyapat Jiampo, Paramat Thimachai, Pimjai Authanont, Suranut Charoensri, Taweesak Wannachalee, Witthawat Naeowong and the ambassadors of Russia Asiiat Aliyeva, Vladisav Belov, Svetlana Vedenskaya, Dina Suliymanova, Natalia Drobotya, Elena Efremova, Nelly Zakirova, Dmitri Kizakozov, Anatoly Kuzin, Tatiana Lipatova, Maria Melnik, Alexander Ovsyannikov, Olga Osipova, Tatiana Pettriko,Aleksey Tarasov, Olga Fedorishina, Alla Khadzegova, Victor Gurevich and Sergey Zubkov and KPL Paris for their contribution to the article.

## Author contributions

**Conceptualization:** Abdulhalim Jamal Kinsara, Hani Sabbour.

**Methodology:** Abdulhalim Jamal Kinsara, Hani Sabbour.

**Validation:** Abdulhalim Jamal Kinsara, Hani Sabbour.

**Writing – original draft:** Abdulhalim Jamal Kinsara.

**Writing – review & editing:** Abdulhalim Jamal Kinsara, Hani Sabbour.
